# Supplementary material for: Peripheral Blood Biomarkers Reveal Dysregulated Monoaminergic Pathways in Obsessive–Compulsive Disorder: A Transcriptional and Epigenetic Analysis
Source: Int J Mol Sci. 2025 Sep 10;26(18):8811. doi: 10.3390/ijms26188811 (PMC12469943; doi:10.3390/ijms26188811)
Supplement: Supplementary file 1 [file ijms-26-08811-s001.zip › ijms-3808033-supplementary.pdf]

Figure S1. Sex-stratified gene expression analysis of *SLC6A4* (a and b), *MAOB* (c and d), and *MB-COMT* (e and f). **p* < 0.05, ***p* < 0.01, ****p* < 0.05, *****p* < 0.0001 Mann–Whitney test.

Figure S2. Sex-stratified DNA methylation analysis at *SLC6A4* (a and b), *MAOB* (c and d), and *COMT* (e and f) gene promoter regions.

Table S1. Overall and sex-stratified gene expression analysis of *SLC6A4*, *MAOB*, and *MB-COMT* in human PBMCs from OCD patients and healthy subjects (CTRL).

Table S2. Groups' means of DNA methylation in human PBMCs from OCD patients and healthy subjects (CTRL) for the individual CpG sites analyzed in *SLC6A4*, *MAOB*, and *COMT* gene promoter regions. Statistically significant differences are highlighted in bold.

Table S3. Sex-stratified groups' means of DNA methylation in human PBMCs from OCD patients and healthy subjects (CTRL) for the individual CpG sites analyzed in *SLC6A4*, *MAOB*, and *COMT* gene promoter regions.

Table S4. Spearman's r and *p* value of correlation between gene expression in human PBMCs from OCD patients and healthy subjects (CTRL). Statistically significant differences are highlighted in bold.

Table S5. Primer sequences used for gene expression analysis with qRT-PCR in PBMCs from OCD patients and healthy individuals.

Table S6. Details of sequences and primers employed for DNA methylation analysis in human PBMCs from OCD patients and healthy controls.

Figure S1.

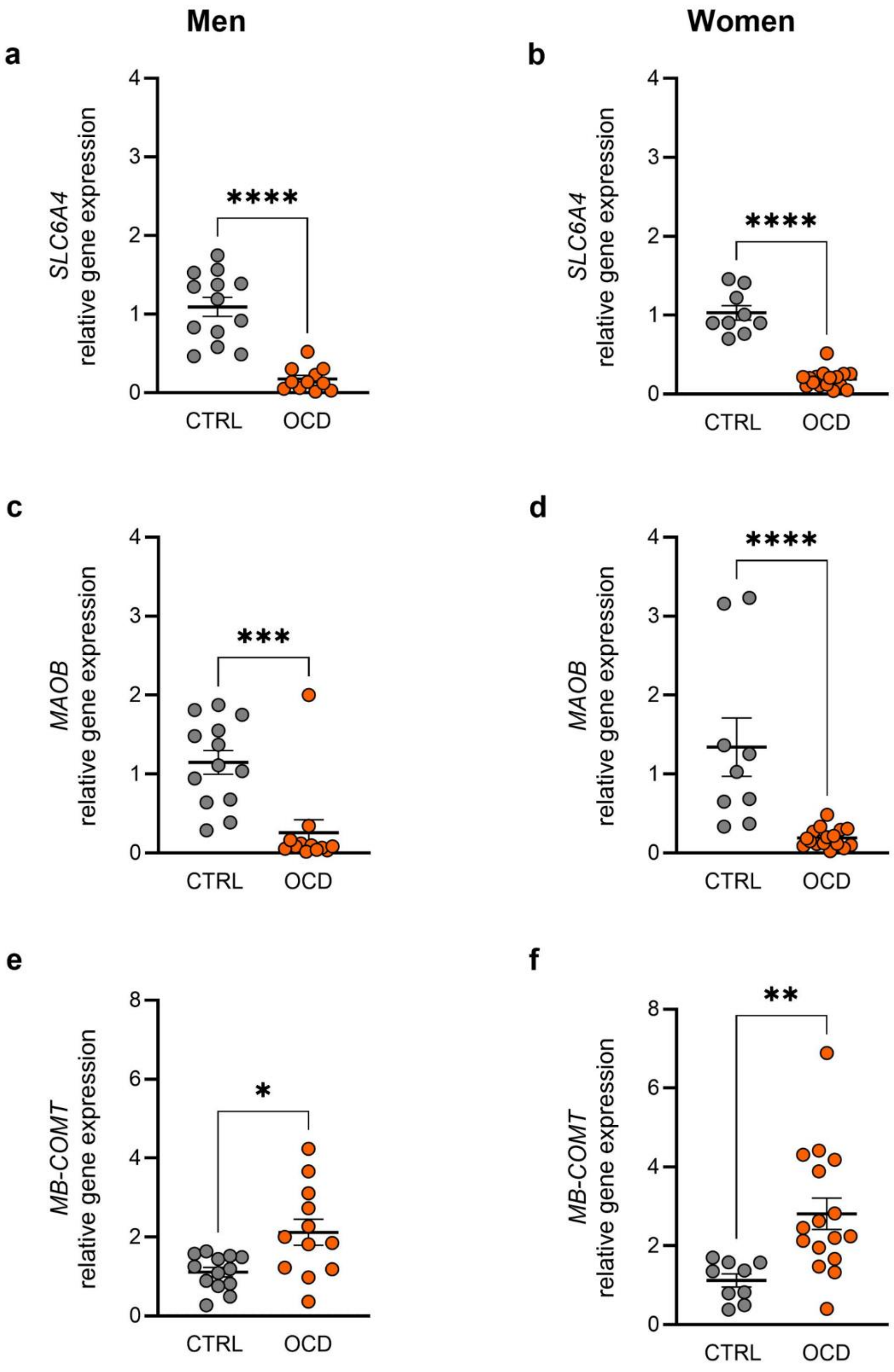
Men
Women
a
b
c
d
e
f
SLC6A4
relative gene expression
MAOB
relative gene expression
MB-COMT
relative gene expression
CTRL
OCD
****
***
*
**
0
1
2
3
4
6
8

Figure S2.

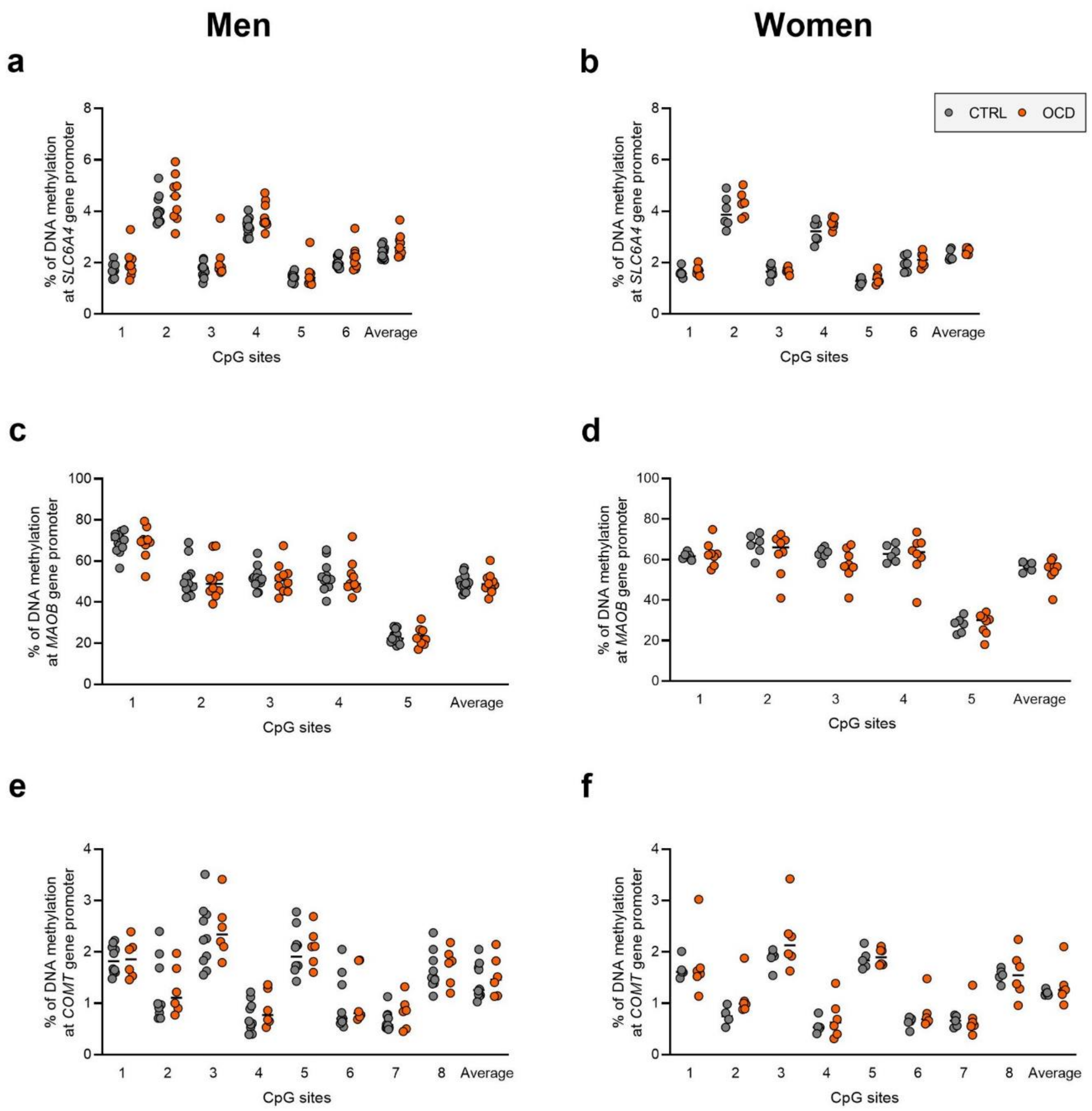

Men
Women
a
b
c
d
e
f
CTRL
OCD
% of DNA methylation at SLC6A4 gene promoter
% of DNA methylation at MAOB gene promoter
% of DNA methylation at COMT gene promoter
CpG sites
Average

Table S1.

	CTRL	OCD	CI	P value
	Mean ± SEM	Mean ± SEM		
		***SLC6A4* relative gene expression**		
**Overall**	1.07 ± 0.08	0.19 ± 0.03	-1.118 to -0.686 (95.06%)	<0.0001
**Men**	1.09 ± 0.12	0.17 ± 0.05	-1.286 to -0.529 (95.26%)	<0.0001
**Women**	1.03 ± 0.09	0.19 ± 0.03	-1.018 to -0.646 (95.06%)	<0.0001
		***MAOB* relative gene expression**		
**Overall**	1.24 ± 0.16	0.14 ± 0.02	-1.519 to -0.666 (95.06%)	<0.0001
**Men**	1.15 ± 0.15	0.26 ± 0.16	-1.445 to -0.555 (95.43%)	0.0002
**Women**	1.34 ± 0.37	0.19 ± 0.03	-1.247 to -0.355 (95.06%)	<0.0001
		***MB-COMT* relative gene expression**		
**Overall**	1.12 ± 0.10	2.45 ± 0.26	0.583 to 1.744 (95.06%)	<0.0001
**Men**	1.11 ± 0.12	2.12 ± 0.33	0.221 to 1.731 (95.43%)	0.0135
**Women**	1.12 ± 0.17	2.81 ± 0.40	0.630 to 2.604 (95.06%)	0.0011

Table S2.

CpG sites	CTRL	OCD	P value
	Mean ± SEM	Mean ± SEM	
***SLC6A4* promoter region**			
Site 1	1.73 ± 0.05	2.00 ± 0.10	0.2344
Site 2	**4.09 ± 0.13**	**4.56 ± 0.15**	**0.0024**
Site 3	1.71 ± 0.06	1.92 ± 0.09	0.5493
Site 4	3.37 ± 0.10	3.64 ± 0.11	0.2340
Site 5	1.40 ± 0.04	1.52 ± 0.08	0.9572
Site 6	2.01 ± 0.06	2.15 ± 0.08	0.9052
Average	2.39 ± 0.05	2.63 ± 0.08	0.3371
***MAOB* promoter region**			
Site 1	66.45 ± 1.13	66.50 ± 0.29	>0.999
Site 2	55.28 ± 2.18	55.71 ± 0.35	>0.999
Site 3	54.12 ± 1.60	54.14 ± 1.04	>0.999
Site 4	53.81 ± 1.70	55.27 ± 0.45	0.9839
Site 5	24.32 ± 0.84	25.20 ± 0.27	0.9990
Average	50.84 ± 1.06	51.36 ± 0.25	>0.999
***COMT* promoter region**			
Site 1	1.81 ± 0.07	1.81 ± 0.15	>0.999
Site 2	1.10 ± 0.16	1.18 ± 0.12	0.9998
Site 3	2.18 ± 0.15	2.35 ± 0.17	0.9407
Site 4	0.69 ± 0.07	0.80 ± 0.11	0.9967
Site 5	1.97 ± 0.10	2.00 ± 0.09	>0.999
Site 6	0.87 ± 0.12	0.97 ± 0.13	0.9992
Site 7	0.67 ± 0.05	0.76 ± 0.09	0.9993
Site 8	1.60 ± 0.08	1.64 ± 0.12	>0.999
Average	1.36 ± 0.08	1.44 ± 0.11	0.9998

Table S3.

CpG sites	CTRL	OCD	P value
	Mean ± SEM	Mean ± SEM	
***SLC6A4* promoter region**			
**Men**			
Site 1	1.72 ± 0.08	2.00 ± 0.19	0.7524
Site 2	4.06 ± 0.16	4.52 ± 0.30	0.2022
Site 3	1.70 ± 0.09	2.04 ± 0.22	0.5369
Site 4	3.43 ± 0.11	3.83 ± 0.17	0.3417
Site 5	1.45 ± 0.05	1.54 ± 0.17	0.9996
Site 6	2.03 ± 0.07	2.22 ± 0.17	0.9626
Average	2.40 ± 0.07	2.69 ± 0.15	0.7109
**Women**			
Site 1	1.61 ± 0.08	1.70 ± 0.08	0.9980
Site 2	3.98 ± 0.26	4.29 ± 0.20	0.4476
Site 3	1.66 ± 0.11	1.69 ± 0.05	>0.9999
Site 4	3.20 ± 0.17	3.53 ± 0.09	0.3652
Site 5	1.27 ± 0.06	1.39 ± 0.10	0.9918
Site 6	1.96 ± 0.13	2.11 ± 0.12	0.9728
Average	2.28 ± 0.08	2.46 ± 0.05	0.9359
***MAOB* promoter region**			
**Men**			
Site 1	66.04 ± 1.41	68.89 ± 2.32	>0.9999
Site 2	50.98 ± 2.19	50.96 ± 3.01	>0.9999
Site 3	51.62 ± 1.41	51.37 ± 2.32	>0.9999
Site 4	52.10 ± 1.89	51.85 ± 2.63	>0.9999
Site 5	23.13 ± 0.87	22.94 ± 1.35	>0.9999
Average	49.38 ± 1.09	49.20 ± 1.60	>0.9999
**Women**			
Site 1	61.66 ± 0.68	62.83 ± 2.16	0.9997
Site 2	67.25 ± 2.20	62.58 ± 3.77	0.7300
Site 3	63.00 ± 1.20	57.43 ± 2.90	0.5490

Site 4	62.98 ± 1.67	61.81 ± 3.71	0.9997
Site 5	27.81 ± 1.56	28.08 ± 1.89	>0.9999
Average	56.54 ± 0.86	54.55 ± 2.26	0.9945
***COMT* promoter region**			
**Men**			
Site 1	1.85 ± 0.09	1.86 ± 0.15	>0.9999
Site 2	1.21 ± 0.19	1.26 ± 0.19	>0.9999
Site 3	2.31 ± 0.19	2.44 ± 0.23	0.9990
Site 4	0.74 ± 0.09	0.90 ± 0.14	0.9966
Site 5	2.00 ± 0.14	2.10 ± 0.16	0.9999
Site 6	0.97 ± 0.16	1.12 ± 0.23	0.9979
Site 7	0.66 ± 0.06	0.82 ± 0.13	0.9958
Site 8	1.63 ± 0.12	1.72 ± 0.15	>0.9999
Average	1.42 ± 0.11	1.53 ± 0.16	0.9998
**Women**			
Site 1	1.67 ± 0.09	1.76 ± 0.26	>0.9999
Site 2	0.75 ± 0.10	1.11 ± 0.16	0.6467
Site 3	1.87 ± 0.09	2.26 ± 0.26	0.4391
Site 4	0.56 ± 0.07	0.71 ± 0.16	0.9971
Site 5	1.86 ± 0.09	1.90 ± 0.07	>0.9999
Site 6	0.63 ± 0.05	0.82 ± 0.14	0.9868
Site 7	0.65 ± 0.05	0.70 ± 0.14	>0.9999
Site 8	1.54 ± 0.06	1.57 ± 0.19	>0.9999
Average	1.20 ± 0.02	1.35 ± 0.16	0.9970

Table S4.

**Interaction**	**Overall**		**CTRL**		**OCD**	
	Spearman's r	P value	Spearman's r	P value	Spearman's r	P value
SLC6A4 x *MAOB*	0.8318	**<0.0001**	0.5065	**0.0162**	0.3437	0.0792
SLC6A4 x *MB-COMT*	-0.4602	**0.0009**	0.2693	0.2255	0.01160	0.9542
MAOB x *MB-COMT*	-0.4177	**0.0028**	0.4907	**0.0204**	0.03603	0.8584

Table S5.

Gene	Gene ID	Primers	Primer sequence	Product size (bp)
SLC6A4	6532	Forward	GGAGACAGGGGTGTGGATAG	156
		Reverse	AAGCTCGTCATGCAGTTCAC	
MAOB	4129	Forward	CGGCATCTCAGGTATGGCA	240
		Reverse	ACATGGTGGATCAGACGCTC	
MB-COMT	1312	Forward	CTGCTTTGCTGCCGAGCTCAGAGGAGAC	127
		Reverse	GCCCAGCAACACAGCTGCCAACAG	
GAPDH	2597	Forward	CAGCCTCAAGATCATCAGCA	106
		Reverse	TGTGGTCATGAGTCCTTCCA	
β-ACT	11461	Forward	GACCCACATCATGTTTGAGACCT	104
		Reverse	CCATCACGATGCCAGTGG	
RPLP0	6175	Forward	TCCTCGTGGAAGGCCCCG	100
		Reverse	TGCCACGCAGGGTTTAAAGA	
COX6A1	1337	Forward	CATCAGGACCAAGCCGTTTCC	164
		Reverse	ATGTGCAGAGTAACGGTCCA	

Table S6.

Gene promoter	Primers	Primer sequences	Position
SLC6A4	Forward	Hs_SLC6A4_01_PM PyroMark CpG assay (PM00065625 - Qiagen)	Chr.17:30235979-30235936
	Reverse		
	Sequencing		
MAOB	Forward	Hs_MAOB_01_PM PyroMark CpG assay (PM00032711 – Qiagen)	Chr.X:43741312-43741256
	Reverse		
	Sequencing		
COMT	Forward	GGGAGTAGTAGGTAGGATGGG	Chr.22:19941650-19941690
	Reverse	TCCCCACCTAAAAAAAAAACTACTT	
	Sequencing	GTAGGTAGGATGGGG	
